# Uncovering the Mechanisms of Intracellular Membrane Trafficking by Reconstituted Membrane Systems

**DOI:** 10.3390/membranes15050154

**Published:** 2025-05-16

**Authors:** Shuhan Chen, Yinghui Liu, Haijia Yu

**Affiliations:** Jiangsu Key Laboratory for Molecular and Medical Biotechnology, College of Life Sciences, Nanjing Normal University, Nanjing 210023, China; 09220607@njnu.edu.cn

**Keywords:** liposome, nanodiscs, vesicle fusion, lipid transfer, membrane trafficking

## Abstract

Intracellular membrane trafficking that transports proteins, lipids, and other substances between organelles is crucial for maintaining cellular homeostasis and signal transduction. The imbalance of membrane trafficking leads to various diseases. It is challenging to uncover the mechanisms of the complicated and dynamic trafficking process at the cellular or animal levels. The applications of functional reconstituted membrane systems, which can mimic the intracellular membrane compartments in a clean and simplified pattern, tremendously facilitate our understanding of the membrane trafficking process. In this review, we summarize applications of the in vitro membrane models, including liposomes, nanodiscs, and single-vesicle platforms, in elucidating molecular mechanisms that govern vesicle fusion and non-vesicular lipid transport, the key steps of membrane trafficking. This review highlights how membrane reconstitution approaches contribute to illustrating the protein-mediated molecular choreography of cellular membranes.

## 1. Introduction

The endoplasmic reticulum (ER), the largest membrane-bound organelle in cells, is essential for lipid biosynthesis in eukaryotic cells. Lipids primarily synthesized in the ER are transported to other membrane-bound organelles or the plasma membrane (PM), maintaining the lipid compositions and functional specificity of the subcellular organelles [1,2]. Another key function of lipid transport is to support organelle biogenesis. For instance, mitochondrial membrane biogenesis relies on the import of most lipids, including phosphatidylserine (PS), phosphatidylinositol (PI), phosphatidylcholine (PC), sphingolipids and cholesterol [3]. The subcellular locations of lipids are critical for their physiological roles. The accurate distribution of lipids requires the lipid transport systems to be tightly regulated [4].

**Figure 1:**
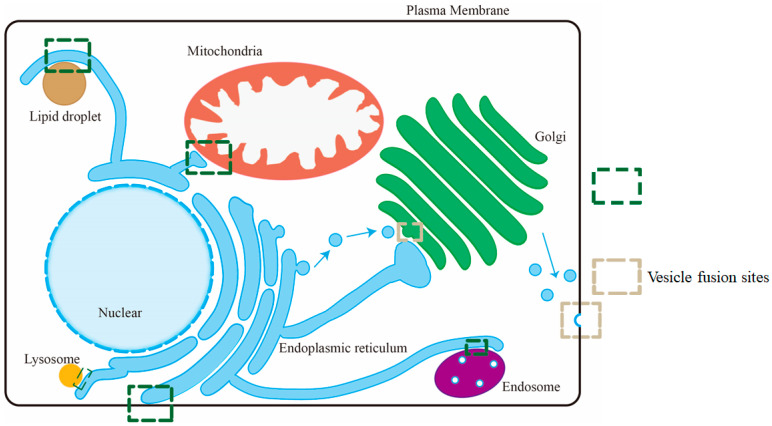
The intracellular membrane trafficking between organelles in mammalian cells. In the vesicular transport pathway, SNARE-dependent membrane fusion occurs at the vesicle fusion sites. The LTP usually mediates lipid homeostasis at membrane contact sites through non-vesicular lipid transport. Examples of membrane contact sites and vesicle fusion sites are shown.

There are two categories of lipid transport pathways in intracellular membrane trafficking: vesicular transport and non-vesicular transport (Figure 1). Vesicular transport involves events including vesicle budding, directional transport, docking, and fusion with the recipient membrane at the vesicle fusion sites, enabling the transport of lipids, proteins, and other vesicle-carrying components between organelles [5,6]. Non-vesicular lipid transport mainly occurs in the physically connected regions between two organelles, known as membrane contact sites (MCSs). The opposite membranes in MCSs are brought closely together, usually within 30 nm. Although vesicular transport provides a large amount of lipids upon fusion, growing evidence suggests that non-vesicular lipid transport mediated by lipid transfer proteins (LTPs) plays a vital role during lipid homeostasis [7]. Non-vesicular lipid transport enables cells to adjust the lipid composition of membranes more precisely than vesicular trafficking, which is particularly advantageous during stress conditions when vesicle fusion is compromised [8]. It can either enrich or remove specific lipids in membranes. This selective modulation of lipid composition not only fine tunes the physical and functional properties of membranes but also regulates the levels of signaling lipids, thereby influencing cellular signaling pathways.

Intracellular membrane trafficking, either vesicular or non-vesicular, is a dynamic and complicated biological process. It is challenging to study membrane trafficking in living cells, which requires the measurement of single lipid movements, due to the limitations of the techniques, e.g., the resolution limit of the light microscope. Artificial membranes or membranes incorporated with purified membrane proteins, such as liposomes, nanodiscs, and supported lipid bilayers (SLBs), have become a valuable resource for understanding the functional and structural aspects of membrane-associated proteins. The reconstituted membrane systems can recapitulate the process of membrane trafficking to some extent and monitor the trace of labeled lipids in vitro. The approach has been successfully applied to investigate a wide variety of membrane protein functions. In this context, membrane reconstitution has been particularly important for examining proteins with membrane trafficking functions. It enables the study of general properties of membrane proteins, such as lipid–protein and protein–protein interactions, protein topology in the membrane, and lipid mixing or exchange between membranes [9].

This review focuses on the application of reconstituted membrane systems in dissecting intracellular membrane trafficking, highlighting how the artificial membrane systems help uncover the mechanisms of soluble N-ethylmaleimide-sensitive factor attachment protein receptors (SNAREs)-mediated intracellular vesicle fusion and LTP-driven lipid transport between organelles (Figure 1).

## 2. The Powerful Reconstituted Membrane Systems Applied in the Research of Intracellular Membrane Trafficking

**Figure 2:**
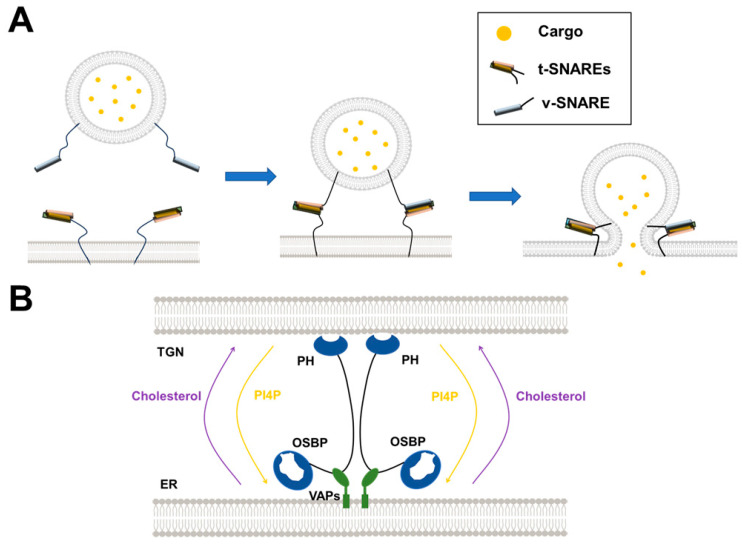
Diagrams illustrating the vesicle fusion and non-vesicular lipid transport. (**A**) During synaptic exocytosis, the t-SNAREs and v-SNARE zipper into the SNARE complex, driving membrane fusion and releasing the cargo. (**B**) OSBP-mediated lipid transport between the ER and TGN.

Vesicle fusion, the last key step of intracellular vesicular transport, involves two membranes approaching and merging into one, releasing the vesicle-carrying cargo. The membranes alone are mutually exclusive, which requires fusion proteins to overcome energy barriers [5]. The SNAREs are the core protein machine of intracellular membrane fusion in mammalian cells. SNAREs are membrane-associated proteins localized to the vesicle (v-SNARE) and the target membrane (t-SNAREs) [10,11]. The v-SNARE pairs with the t-SNAREs to form a four-helix trans-SNARE complex initiating membrane fusion [12,13,14]. Synaptic vesicle fusion is the most extensively studied membrane fusion pathway (Figure 2A). In the non-vesicular lipid transport, the membranes from two organelles are tethered in a short distance, where the LTPs execute the transfer of bulk or specific lipids without membrane fusion [15,16]. For example, Oxysterol Binding Protein (OSBP) can transport PI4P and cholesterol between the ER and TGN (Figure 2B).

While both vesicle fusion and non-vesicular lipid transport contribute to lipid exchange and supplementation, only vesicle fusion transports non-lipid components, including membrane proteins and interior contents. Beyond their biological significance, membrane fusion holds promise for drug delivery, offering a faster and more efficient alternative to conventional endocytic pathways. Lipid vesicles (or liposomes) have been favored for transporting various cargoes into cells, such as small molecules, proteins, and nanoparticles. Unlike vesicle fusion, LTP-mediated lipid transport can usually sense and transfer specific types of lipids, which is critical for lipid homeostasis and lipid signal transduction [17]. Multiple powerful reconstituted membrane systems built in vitro have been applied to study vesicle fusion and non-vesicular lipid transport. The reconstituted membrane fusion systems will help identify SNARE pairs in new fusion pathways, e.g., the exosome secretion, and reveal the precise regulation of membrane fusion by SNAREs and their regulatory factors. The lipid transport systems have become a standard method to validate a new LTP and its transferred lipid species.

### 2.1. The Different Types of Liposomes Employed in the Reconstituted Membrane Systems

#### 2.1.1. Liposomes and Proteoliposomes

Liposomes were initially identified by Bangham A. D. and his team in 1964 [18]. Liposomes are colloidal spherical structures formed through the self-assembly of amphiphilic lipid molecules, such as phospholipids, in a solution. The liposomal membrane can consist of one or more lipid bilayers arranged around an internal aqueous core, with the polar head groups oriented towards the inner and outer aqueous phases [19]. This well-organized structure endows liposomes with the unique capacity to load and deliver molecules of varying solubility. Hydrophilic molecules can be accommodated in the internal aqueous core, hydrophobic ones within the lipid bilayer, and amphiphilic molecules at the water/lipid bilayer interface. In the early 1970s, proteoliposomes were initially created by incorporating the Ca^2+^-dependent ATPase from the sarcoplasmic reticulum into nano-sized liposomes [20]. In the late 1980s, proteoliposomes with the sarcoplasmic reticulum K^+^ channels were made using cell-sized liposomes (around 10 μm in diameter) to measure single ion channel currents [21].

There are several methods to make liposomes from the lipid mixtures. As for membrane fusion, it is critical to reconstitute membrane proteins topologically into liposomes. If a protein has a large hydrophobic domain, moving it from the micellar system into the liposomes is difficult, even with low detergent concentrations. In such cases, the most frequently used reconstitution method is the co-solubilization of membrane proteins and lipids. This approach applies detergent to extract and purify proteins from other natural biomembrane components and keeps the membrane protein stable. Then, a lipid suspension with the same detergent is added. After incubation, the detergent is removed using a proper technique, e.g., dialysis, gel filtration, or hydrophobic resins. Once the detergent is gone, lipid molecules tend to form a bilayer structure, isolating their hydrophobic tails from the aqueous medium. At the same time, membrane proteins are incorporated among the lipid groups, creating lipid–protein domains and forming proteoliposomes. Diverse proteoliposome systems can be obtained by altering the lipid species and proportions, incubation time, detergent removal method, or the speed of detergent removal, differing in the type and amount of reconstituted proteins [22,23].

Over the past decade, proteoliposomes have become significant tools for studying lipid–protein interactions and many other biotechnological applications. Compared to natural membrane systems, proteoliposomes require fewer lipidic and protein components, simplifying the design and interpretation of experiments. However, a drawback is the need for methodological optimization when each new specific protein is reconstituted.

#### 2.1.2. GUVs, LUVs and SUVs

Giant unilamellar vesicles (GUVs), with sizes up to 100 μm, are synthetic vesicles formed by free-standing bilayers that trap an isotonic solution [24]. They are typically produced by simply rehydrating SLBs [25]. When dried SLBs are rehydrated under an electric field and with isotonic solutions, it leads to high yields of giant vesicles with adjustable sizes [25]. GUVs can also be prepared by other methods, e.g., the polymer-assisted swelling on an agarose gel [26]. The diameters of GUVs are usually between 10 and 100 μm, averaging 20 μm [27]. Because of their free-standing structure, soft exteriors, geometry, and size range, GUVs are great models for studying membrane processes in 3D cell–cell interfaces [28]. The geometry and large size of GUVs make them attractive models, as they are easily accessible via optical microscopy. Moreover, bilayer behaviors, such as clustering, liquid–liquid phase separation, and vesicle-to-cell interactions, can easily be reproduced by GUVs in vitro [29]. For example, GUVs have become a valuable tool in examining the presynaptic active zone organization driven by phase separation [30]. We recently utilized the GUV model to investigate the phase separation of synaptotagmin-1, an essential Ca^2+^ sensor in synaptic vesicle fusion [31]. Ilya Bezprozvanny’s group took GUVs as an in vitro model membrane to study the organization of the Sigma 1 receptor, a key component of ER–mitochondrial contact sites [32].

Liposomes, including small unilamellar vesicles (SUVs) of 50–300 nm and large unilamellar vesicles (LUVs) of 200–1000 nm, are made by breaking multilamellar liposomes via methods such as extrusion, sonication, homogenization, or detergent dialysis, with extrusion being the most efficient for generating homogeneous SUVs [33,34,35]. They are powerful for reconstituting lipid/protein scaffolds, studying cellular processes, such as ion channels, membrane fusion, and lipid transport, and serving as drug/vaccine delivery platforms.

### 2.2. The Liposome-Based Assays for the Measurement of Vesicle Fusion

#### 2.2.1. Liposome–Liposome Lipid Mixing

As a key part of intracellular membranes, lipids are essential to numerous cellular activities. The lipid composition of different organelles and organelle-generated vesicles varies, which is closely related to their biological functions. The in vitro membrane reconstitution offers the free design and definition of lipid composition in protein-free liposomes or proteoliposomes. The membrane fusion occurs through the lipid mixing of two membrane compartments. Thus, a liposome-liposome lipid mixing assay was developed and has been widely used to monitor the dynamics of membrane fusion (Figure 2A) [34,36].

**Figure 3:**
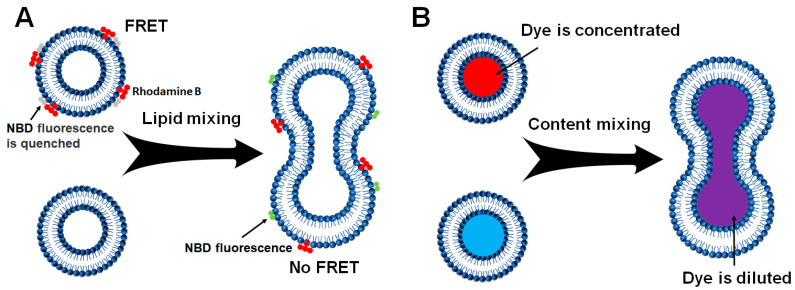
Diagrams showing the liposome-liposome lipid mixing assay and liposome-liposome content mixing assay. The reconstituted proteins upon each requirement are not shown for clarity. (**A**) Before fusion, the NBD fluorescence is quenched (gray) by rhodamine (red) in the NBD/rhodamine-labeled liposomes. When the labeled liposomes fuse with unlabeled liposomes, NBD fluorescence (Green) increases due to the diminished NBD-rhodamine FRET. (**B**) Unlabeled liposomes (blue) are mixed with sulforhodamine B-loaded liposomes (red) in which the sulforhodamine B fluorescence is inhibited by self-quenching. The fusion of the liposomes (purple) leads to mixing their contents and dequenching sulforhodamine B fluorescence.

A standard liposome-liposome lipid mixing fusion reaction is usually based on the Fluorescence Resonance Energy Transfer (FRET) and contains donor and acceptor liposomes, which can be conducted in a microplate [34]. The donor liposomes containing nitrobenzoxadiazole (NBD)-labeled lipids quenched by Lissamine Rhodamine B-labeled lipids were directed to fuse with unlabeled acceptor liposomes. Before fusion, the excitation of NBD fluorescence predominantly transfers energy to rhodamine molecules rather than direct photon emission. Subsequent membrane fusion results in fluorophore dilution, reducing FRET efficiency and leading to NBD fluorescence recovery through dequenching (Figure 3A) [37]. An increase in NBD fluorescence can be employed to measure the fusion events. At the end of the fusion reaction, excess detergent, such as 3-[(3-cholamidopropyl)dimethylammonio]-2-hydroxy-1-propanesulfonic acid (CHAPSO) or dodecylmaltoside (DM), is added to the liposomes. The maximum fluorescence change can be obtained to calculate the fusion rate.

#### 2.2.2. Liposome-Liposome Content Mixing

A real membrane fusion event is marked by the merger of aqueous contents without leakage, while lipid mixing assays may track liposome positioning, aggregations, and lipid exchange instead of actual fusion [38,39,40]. As lipid mixing cannot differentiate hemifusion from full membrane fusion, it is possible that membrane fusion does not go beyond the hemifusion stage. For this purpose, dithionite treatment, which selectively abolishes the NBD fluorophores on the outer leaflets of the liposomes, can detect the inner-leaflet fluorescence in the lipid mixing assay [39].

Alternatively, we can use a liposome-liposome content mixing assay with donor liposomes encapsulated fluorescence dye, such as sulforhodamine B or calcein, at self-quenching concentrations and fuse them with the empty acceptor ones (Figure 2B) [39,40,41,42,43]. Upon fusion, content mixing between donor and acceptor liposomes leads to the concentration dilution of fluorescence dye (Figure 3B). The fluorescence dequenching is then measured to indicate fusion extent and rate. Excess detergent (such as CHAPSO and DM) at the end of each reaction can be applied to lysis the liposomes. The maximum fluorescence change can be obtained to calculate the fusion rate of content mixing.

#### 2.2.3. Liposome-Nanodisc Lipid Mixing and Content Mixing

**Figure 4:**
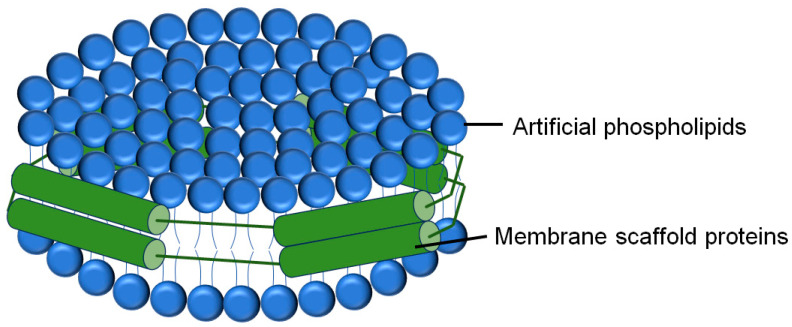
The structure model of a nanodisc: a planar phospholipid bilayer wrapped by MSPs. Membrane proteins can be reconstituted into the lipid bilayers. The protein barrier completely blocks the aqueous solution from the hydrophobic lipid chains. We included lines between protein domains to allow for visualization of the hydrophobic tails within the nanodisc.

The above two methods are all based on liposomes with high membrane curvature. Using nanodisc technology, Stephen G Sligar’s group pioneered the first attempt to reconstitute membrane proteins in planar phospholipid bilayers [44]. The nanodisc is a self-assembled structure that can be used to reconstitute integral membrane proteins into phospholipid bilayers. Typically 8–16 nm in diameter, nanodiscs consist of a discoidal phospholipid bilayer surrounded by a protein belt formed by two helical membrane scaffolding protein (MSP) molecules (Figure 4) [45]. MSPs, derived from human serum apolipoprotein A1, are peptides usually made of 22 residues that form proline and glycine helical repeats. The stability of membrane proteins within nanodiscs is attributed to the strong interaction between the MSP’s hydrophobic residues and the lipid acyl chain [46].

The potential of nanodiscs is demonstrated by their utility in diverse biochemical and biophysical measurements, including high-resolution NMR [47,48], single-molecule fluorescence experiments [49,50], and cryo-EM structure analysis [51,52]. Importantly, these methods can be adapted to accommodate membrane proteins in the 2-D planar lipid bilayers. Incorporation of a membrane protein into a lipid nanodisc requires initial solubilization of the protein with detergent [53]. There are two common ways to reconstitute membrane proteins into nanodiscs: the crude solubilized protein can be purified first and then reconstituted or directly incorporated into nanodiscs without pre-purification. A key advantage of the latter is that it can be used for membrane proteins unstable in detergent, allowing affinity purification after the target has been reconstituted in nanodiscs. In most cases, the membrane proteins purified in detergent are used to prepare nanodiscs. The native lipids are removed during the protein purification, simplifying the determination of the accurate calculation of the MSP to phospholipid ratio, which is important for the preparation of nanodiscs [53].

In a standard protocol of reconstituted nanodisc–liposome fusion reactions, lipid mixtures were resuspended in a reconstitution buffer containing proper concentrations of detergent, together with MSPs and other proteins that need to be reconstituted. Nanodisc self-assembly occurs after removing the detergent via dialysis or SM-2 Bio-Beads adsorption [41,54,55]. As a planar membrane, lipid nanodisc is usually set as the acceptor in the liposome-nanodisc membrane fusion assays. The donor can be either the FRET pair-labeled lipid mixing liposomes or fluorescence dye-encapsulated content mixing liposomes, as described above. The fusion reactions are performed similarly to the procedures of liposome-liposome lipid mixing or liposome-liposome content mixing assay [41,54,55].

#### 2.2.4. Single-Vesicle Fusion

**Figure 5:**
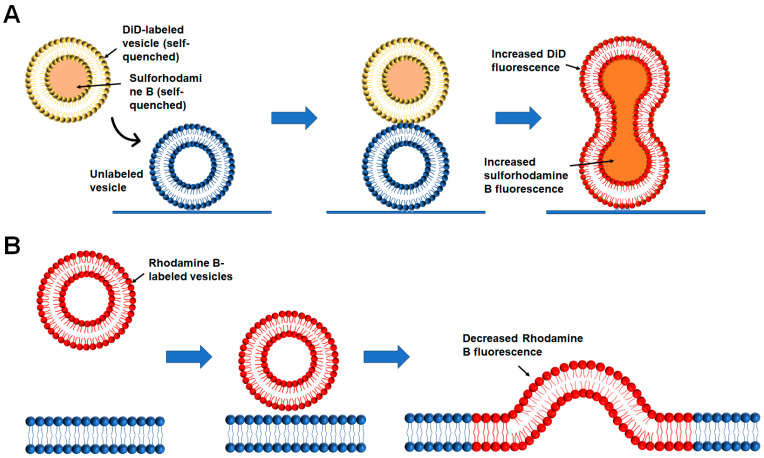
Diagrams showing the single-vesicle fusion assays. (**A**) Single vesicle-vesicle fusion. The reconstituted proteins are not shown. Donor vesicles labeled with self-quenched sulforhodamine B (content dye) and 1,1′-dioctadecyl- 3,3,3′,3′-tetramethylindodicarbocyanine perchlorate (DiD, lipid dye) are added to the chamber containing acceptor vesicles immobilized on a surface via biotin-neutravidin interactions. Single-vesicle content mixing and lipid mixing events can be monitored by the increased fluorescence intensity of sulforhodamine B and DiD, respectively. This assay can discriminate hemifusion and complete fusion and is used to measure Ca^2+^-triggered synaptic vesicle fusion. (**B**) Single vesicle–planner membrane fusion. The proteins are not shown for clarity. Supported bilayers were perfused with vesicles containing 1 mol % Rhodamine-B-dioleoylphosphatidylcholine. Fusion events exhibit a fast drop of the mean Rhodamine B fluorescence intensity because of polarization changes, followed by lipid diffusion from a spherical vesicle into the supported planar bilayer.

The liposome-based bulk fusion assay is powerful and relatively easy to manipulate. However, it is hard to distinguish the detailed fusion stages, such as docking, hemifusion, and complete fusion. A single-vesicle fusion assay using content and lipid-mixing indicators was then developed to meet these requirements (Figure 5A) [56,57,58,59]. Single-vesicle fusion experiments have been designed to capture individual fusion events and study fusion intermediates and kinetics. In these experiments, one vesicle with t- or v-SNAREs is fixed on a glass substrate via PEG/PEG-biotin-neutravidin. The other vesicle with complementary SNAREs is added to the solution. For example, the assay developed by Yoon et al. has immobilized t-vesicles tethered to a passivated surface via neutravidin-biotin linkages, and free v-vesicles with reconstituted v-SNAREs and synaptotagmin are then added to the reaction solution [60,61]. Then, the content and lipid mixing between t- and v-vesicles can be measured using TIRF microscopy. In a subsequent study, this method was employed to investigate the impact of synaptotagmin and complexin on membrane fusion between individual vesicles [60,61,62].

Domanska et al. observed SNARE-dependent fusion of single reconstituted vesicles to planar target membranes within a few milliseconds, nearing the speed of Ca^2+^-triggered synaptic fusion (Figure 5B) [63]. A major advantage is that vesicle docking and fusion can be separately distinguished and analyzed in these single-vesicle fusion assays. Moreover, excellent control over the diffusion of the reconstituted fusion proteins can be achieved in appropriately prepared planar target membranes. These assays have become effective in clarifying the specific mechanistic roles of various regulatory proteins in calcium-regulated neuronal exocytosis in recent years [64].

### 2.3. FRET-Based Lipid Transport

Membrane fusion represents a fundamental mechanism that integrates two distinct bilayers, resulting in the homogenization of the lipid components [65,66]. In contrast, the non-vesicular lipid transport can occur between membranes at short distances, where lipids are extracted by LTPs from one membrane and diffused into another. Therefore, the LTP-mediated lipid transport contributes to either the non-selective bulk lipid exchange or the heterogeneity of organelle membranes by regulating specific lipid levels [67,68].

**Figure 6:**
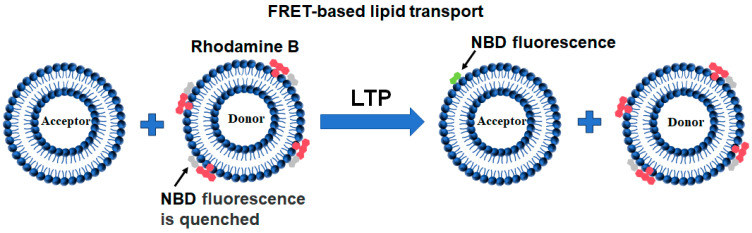
Diagrams showing the FRET-based lipid transport assay. Prior to the lipid transport reaction, NBD fluorescence from the donor liposomes was quenched (gray) by neighboring rhodamine molecules (red). The transport of fluorescence-labeled lipids leads to the dequenching of the NBD fluorescence (green).

The FRET-based lipid transport assay is the most commonly used in vitro biochemical method to measure LTP-mediated lipid transfer [35]. It is modified from the liposome-liposome lipid mixing assay developed in the study of membrane fusion. In this assay, the lipid to be measured in the donor liposomes is labeled by NBD fluorophore either through the synthesized NBD-labeled lipid (e.g., NBD-labeled glycerolipids or cholesterol) or the lipid-bound NBD-labeled protein fragment (such as the labeling of PS or PI4P) [69,70]. Prior to the lipid transfer reaction, NBD emission from the donor liposomes was quenched by the neighboring rhodamine molecules through FRET (Figure 6). The transfer of fluorescence-labeled lipids from the donor to the acceptor liposomes results in the dramatic dequenching of the NBD fluorescence.

## 3. Uncovering the Mechanisms of SNARE-Mediated Intracellular Vesicle Fusion by Reconstituted Membrane Systems

Many cellular processes, such as hormone and neurotransmitter release, depend on membrane fusion. In mammalian cells, membrane fusion is synergistically regulated by SNAREs and other regulatory factors [71,72]. For example, neurotransmitter release is triggered by Ca^2+^ influx from action potentials, leading to the fusion of synaptic vesicles with the presynaptic PM, facilitated by multiple synaptic proteins, including SNAREs, synaptotagmin, complexin, Munc13-1, and Munc18-1 [11,73,74,75]. The imbalance of membrane fusion leads to various diseases, such as developmental disorders, diabetes, and Parkinson’s disease [76,77,78]. Uncovering how membrane fusion is executed will provide a foundation for understanding the physiology of vesicle fusion and related diseases.

It is well known that the SNAREs are the minimal fusion machinery that provides the energy to overcome the repulsion between membranes in intracellular membrane fusion (Figure 2A) [5,79]. The complementary SNAREs zipper from the N-termini to membrane-anchored C-termini, forcing the apposed membranes to fuse, transferring cargo from the vesicle to the target organelle lumen. SNARE proteins dynamically assemble and disassemble during vesicle fusion. The assembly of the SNARE complex is required to drive vesicle fusion. After fusion, SNAREs are disassembled by alpha soluble N-ethylmaleimide-sensitive factor attachment protein (α-SNAP) and N-ethylmaleimide-sensitive factor (NSF), preparing for the next round of fusion [80,81]. The assembly and disassembly processes are further regulated by other regulators, which is critical for accurate spatiotemporal control [82,83,84]. It is necessary to develop compositionally defined reconstituted membrane systems using purified protein and lipid components to uncover the complicated mechanisms of intracellular vesicle fusion. We take the following three membrane fusion processes, synaptic vesicle fusion, GLUT4 vesicle fusion, and autophagosome–lysosome fusion, as examples to discuss the contributions of in vitro reconstituted membrane systems in the membrane fusion field.

### 3.1. Synaptic Vesicle Fusion

The reconstitution of vesicle fusion using purified synaptic proteins and lipids has played a central role in understanding the synaptic exocytosis mechanism. Many vital discoveries in this area are revealed through the reconstitution of neuronal SNARE-mediated fusion, which is accountable for synaptic exocytotic activity in releasing neurotransmitters into the synaptic cleft between neurons. The primary neuronal SNAREs consist of one helix each from Syntaxin-1 and VAMP2 (synaptobrevin), while SNAP-25 contributes the other two helices to complete the bundle [11,73,74,75]. Weber and colleagues integrated SNAREs into vesicles made of artificial lipid bilayers [79]. They demonstrated that donor vesicles reconstituted with VAMP2 could fuse with acceptor vesicles containing Syntaxin-1 and SNAP25 [79]. van den Bogaart et al. used liposome-liposome lipid mixing and content mixing experiments to demonstrate that one copy of the SNARE complex is sufficient for membrane fusion [42]. Lei Shi et al. took advantage of liposome-nanodisc lipid mixing and content mixing assays, confirming that one SNARE complex can fuse, further showing three SNARE complexes are required to keep the nascent fusion pore open [54,55].

While SNAREs drive fusion thermodynamically, the fusion kinetics must be controlled tightly to meet physiological requirements. Benefiting from in vitro membrane reconstitutions, a variety of regulators have been identified as fusion accelerators or clampers. Munc18-1 is a member of the Sec1/Munc18-1 (SM) family that is required for all the intracellular SNARE-mediated membrane fusion [5]. It is suggested that SNARE complex assembly is initiated by a binary complex of Munc18-1 with the closed conformation of Syntaxin-1, which hinders the formation of the SNARE complex with SNAP-25 and VAMP2. Reconstitution experiments revealed that Munc13-1 can mediate the transition from the Munc18-1-closed syntaxin-1 binary complex to the SNARE complex [85]. In addition, Munc18-1 and Munc13-1 offer protection of the SNARE complex from the disassembly of NSF and αSNAP before the fusion [83,84,85]. Munc18-1 interacts with t-SNAREs through its domain 3a SNAER-like peptide (SLP) to promote SNARE-dependent membrane fusion [86,87]. Another important application of membrane reconstitution in synaptic vesicle fusion is the dissection of the Ca^2+^-stimulated fast fusion. Synaptotagmin-1, the primary Ca^2+^ sensor of neurotransmission, can be either reconstituted into the v-SNARE vesicles or added to the fusion reactions using its cytosolic domain [85,88,89]. In the liposome-liposome lipid mixing and content mixing assays, synaptotagmin-1 stimulates SANRE-dependent membrane fusion in response to Ca^2+^ [85,88,89]. Interestingly, synaptic vesicles rapidly fuse with properly reconstituted t-SNARE membranes. In the hybrid in vitro single-vesicle fusion assays, the synaptic vesicles are close to the physiological state in cells and may offer advantages for examining the precise SNARE-mediated fusion mechanisms on the target membrane [90]. Furthermore, the single-vesicle fusion assays, measuring both content and lipid mixing, provide further detailed information for the Ca^2+^-triggered synaptic vesicle fusion, other than the bulk liposome fusion assays [61,62,63].

### 3.2. GLUT4 Vesicle Fusion

Glucose transporter 4 (GLUT4) is a transmembrane protein mainly expressed in adipocytes and skeletal muscles. Insulin-controlled GLUT4 trafficking is central to maintaining blood glucose homeostasis [91]. Under the basal state, GLUT4 resides primarily in the intracellular compartments. When insulin levels rise after meals, GLUT4 translocates from intracellular GLUT4 storage vesicles (GSVs) to the PM.

**Figure 7:**
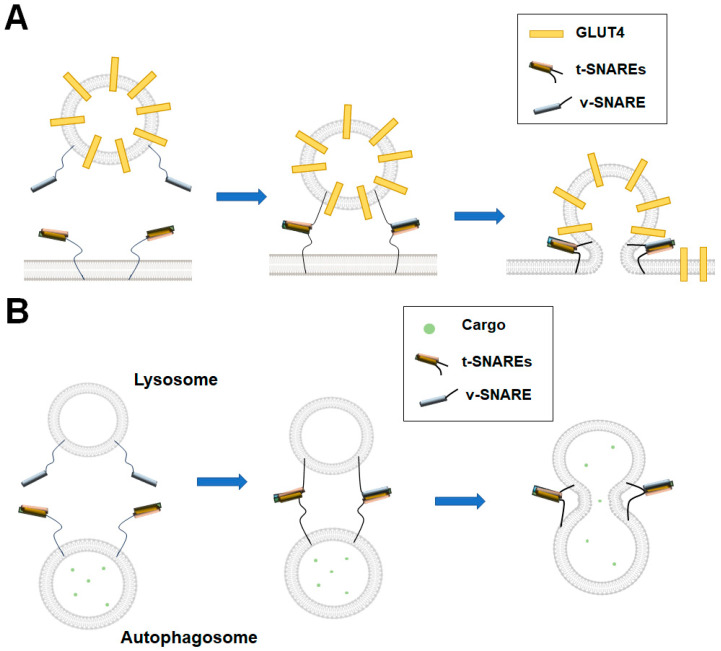
Diagrams showing the GLUT4 vesicle fusion and autophagosome–lysosome fusion. (**A**) In GLUT4 exocytosis, the t-SNAREs on the PM and v-SNAREs in the vesicles assemble into the SNARE complex, driving membrane fusion. GLUT4 is then translocated to the PM. (**B**) The t-SNAREs and v-SNARE assemble into the SNARE complex to mediate the autophagosome–lysosome fusion. The cargoes carried by two organelles mix after fusion.

Similar to synaptic vesicle fusion, the fusion of GSVs with PM also involves SNAREs and their regulators (Figure 7A). However, the cognate SNAREs mediating GLUT4 vesicle fusion differ from synaptic vesicle fusion [91]. In GLUT4 vesicle fusion, syntaxin-4 and SNAP-23 constitute the t-SNAREs, whereas VAMP2 serves as the major v-SNARE [91]. We reconstituted the GLUT4 t-and v-SNAREs into proteoliposomes and investigated the regulation of insulin-stimulated GLUT4 exocytosis [34]. Using the in vitro reconstituted liposome lipid mixing and content mixing experiments, we discovered that Munc18c promotes trans-SNARE assembly to activate membrane fusion, while Doc2b stimulates GLUT4 vesicle fusion in a Ca^2+^-dependent manner [39,40,92].

### 3.3. Autophagosome–Lysosome Fusion

Autophagy is a degradative pathway that contributes to cell homeostasis in normal and under stress conditions. It is established that autophagy can be a highly selective process that serves to specifically degrade protein aggregates, organelles (mitochondria, peroxisomes, endoplasmic reticulum), and pathogens [93]. Basal levels of autophagy are independent of external stimuli, such as starvation or oxidative stress, and are important in maintaining cell homeostasis [94]. After the formation of the autophagosome, the fusion of the autophagosome and lysosome occurs to deliver cargo into the lysosome (Figure 7B).

In vitro reconstitution experiments have been applied to study the autophagosome–lysosome fusion, the late autophagy step. The SNARE complex, composed of syntaxin-17, SNAP-29 and VAMP8, drives efficient membrane fusion in the in vitro reconstituted liposome lipid or content mixing assays [95]. ATG14 stabilizes the SNARE complex and enhances its membrane fusion activity [95]. Mechanistically, ATG14 alone can mediate liposome tethering and assist SNARE-mediated membrane fusion [95]. Another study showed that a VAMP8 phosphorylation mimic mutant inhibits autophagosome–lysosome fusion in vitro, and dephosphorylated VAMP8 facilitates the recruitment of SCFD1, a Sec1/Munc18-like protein, to autolysosomes [96,97].

## 4. Dissecting the LTP-Mediated Lipid Transport at MCSs by Reconstituted Membrane Systems

More and more LTPs, which transfer lipids at MCSs, have been discovered in the past few years. They exist extensively in every species, ranging from bacteria to animals. According to their structural similarity and sequence homology, LTPs are categorized into 27 distinct protein families (The table of LTP families was reported in a previously published review article by Louise H Wong et al.) [17]. A key feature of LTPs lies in their capacity to create a hydrophobic environment for lipids, resulting in a significant drop in the free energy level of lipids when compared with that in an aqueous solution [17]. Based on how they transfer lipids, LTPs are typically split into two main groups: shuttle-type LTPs and bridge-type LTPs, which assist lipids in crossing the hydrophilic cytoplasmic regions between MCSs. Each type of LTP is capable of generating a hydrophobic cavity to house lipids. When lipids traverse the cytoplasm, this cavity shields them, even though the cavity size differs across LTP types [68,98,99]. The lipid transfer activity and selectivity of multiple LTPs have been studied by the in vitro reconstituted membrane systems, such as Extended synaptotagmins (E-Syts) and vacuolar protein sorting 13 (VPS13), and OSBP and its related proteins (ORPs).

### 4.1. LTPs Without Apparent Lipid Selectivity

**Figure 8:**
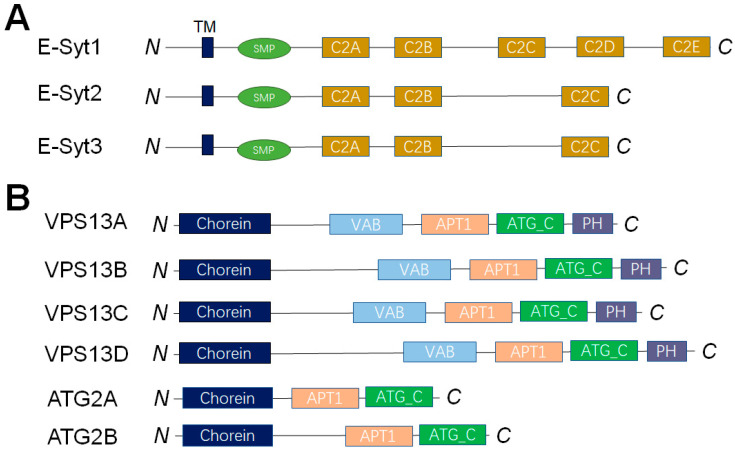
Diagrams of the protein families of E-Syts (**A**) and VPS13 (**B**). The major functional domains are shown in each of the proteins.

E-Syts are integral membrane proteins located at the ER. They are conserved proteins with an N-terminal membrane anchor followed by a synaptotagmin-like mitochondrial lipid binding protein (SMP) domain and multiple C2 domains (Figure 8A). As a family of SMP domain proteins, E-Syts are considered as LTPs at ER-PM, ER–peroxisome, or ER–lipid droplet–mitochondria contact sites [100,101]. The crystal structure showed that the SMP domain forms a dimer that harbors glycerolipids without selectivity [68]. By the in vitro reconstituted membrane systems, E-Syt1 was demonstrated as a Ca^2+^-dependent LTP, which transfers glycophospholipids and DAG bidirectionally [69,102]. The length of the SMP dimer is around 9 nm, which is much shorter than the average distance between ER and PM. The “shuttle model” was further supported by an in vitro reconstituted assay using the DNA-origami nanostructures [103,104]. We employed the in vitro liposome-based lipid transfer assays to systematically investigate the mammalian E-Syt1, yeast Tcb3, and *Arabidopsis* synaptotagmin 1 [69,105,106]. Our data indicated that membrane tethering and lipid transport are the conserved functions of E-Syt proteins, although each has divergent mechanisms. A recent in vitro reconstitution-based study showed that the SMP domain of E-Syt transfers lipids efficiently through its tip region to recognize the curved subdomain of tubular ER and the acidic-lipid enriched PM [107].

The large and highly conserved VPS13 proteins are demonstrated as lipid transporters that function at MCSs. The mutations of the four VPS13 isoforms, which are located at different MCSs, are all connected to neurological disorders: chorea-acanthocytosis (VPS13A), Cohen syndrome (VPS13B), early onset Parkinson’s disease (VPS13C) and ataxia (VPS13D) [7,108,109,110]. VPS13 and the bridge-like LTP superfamily it belongs to, including ATG2, have attracted great interest recently (Figure 8B). A critical characteristic of VPS13 and ATG2 is that they can bridge gaps between organelles at a distance of 15–30 nm. The N-terminal tubular region of VPS13 or ATG2 contains a hydrophobic cavity that can hold and transport glycerolipids directly at the MCSs. By the in vitro liposome-based lipid transfer assay, the lipid transfer functions of VPS13 and ATG2 have been directly measured [111,112]. Both VPS13A and ATG2 transport glycerolipids between membranes robustly without apparent selectivity [111,112].

### 4.2. The OSBP/ORPs Family with Lipid Selectivity

OSBP was discovered as a protein with a strong affinity for oxysterols [113]. OSBP and ORPs comprise a functionally conserved LTP family in eukaryotes, termed the OSBP/ORPs protein family, which is characterized by a conserved OSBP-related domain (ORD) that contains a hydrophobic pocket harboring specific lipids, such as cholesterol, PS, and PI4P [114,115,116]. Most OSBP/ORPs possess dual-membrane-targeting domains, positioning them across MCSs and facilitating lipid transport between organelle membranes. Beyond mediating MCS formation [117], they can sense lipid dynamics and transport lipids [118,119], thus playing roles in lipid metabolism and signal transduction [120,121].

**Figure 9:**
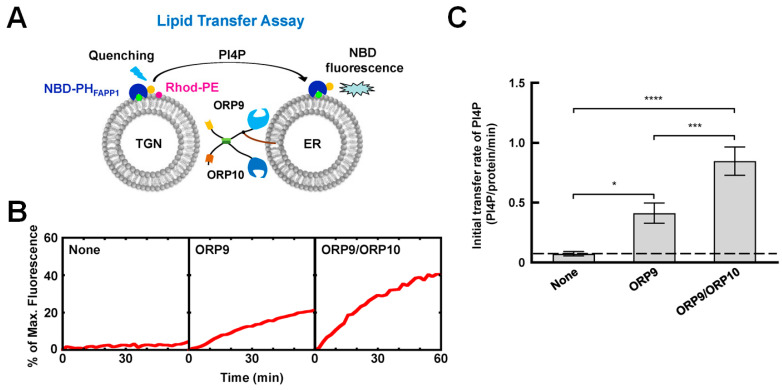
ORP9 and ORP10 transport PI4P in the in vitro reconstituted membrane system. (**A**) Diagram of the FRET-based lipid transport assay. Before the reaction, NBD fluorescence from the TGN-like liposomes was quenched by rhodamine molecules through FRET. The transfer of fluorescence-labeled lipids results in the dequenching of the NBD fluorescence. (**B**) Lipid transport of the liposomes in the absence or presence of ORP9 and ORP9/ORP10. The data are presented as the percentage of maximum fluorescence change. (**C**) Initial lipid transfer rates of PI4P in the reactions shown in B. Error bars indicate standard deviation. p values were calculated using one-way ANOVA with Tukey’s multiple comparisons test. * *p* = 0.0140. *** *p* = 0.0004. **** *p* < 0.0001. Adapted with permission from reference [70].

OSBP, a well-studied member of the OSBP/ORPs family, contains the FFAT motif that interacts with the ER-anchored vesicle-associated membrane protein-associated proteins (VAPs) and a PI4P-binding pleckstrin homology (PH) domain. Mesmin et al. [67] demonstrated that OSBP can tether two types of GUVs in vitro: one population marked with VAP and another containing PI4P, mimicking the formation of MCSs. OSBP is capable of countertransporting PI4P/cholesterol at ER–Golgi MCSs and transporting cholesterol from the ER to lysosomes. In addition to OSBP, ORP9, ORP10 and ORP11, Golgi MCSs are also located at the ER. While ORP9 is anchored to the ER through its FFAT domain, ORP10 and ORP11 can not directly target the ER. However, they can target the ER by forming heterodimers with ORP9 [70,122,123]. The in vitro liposome-based lipid transport studies showed that ORP9/ORP10 robustly transfer PI4P (Figure 9) [70]. Interestingly, they can countertransport PS but not cholesterol [70,122,123]. A recent study reported that OSBP, ORP9, ORP10 and ORP11 are recruited into damaged lysosomes to repair damaged membranes [122].

ORP1 has two isoforms: a shorter variant called ORP1S, which primarily consists of an ORD, and a longer variant called ORP1L, which includes additional domains such as an FFAT motif, a PH domain, and three ankyrin repeats near its amino terminus [124]. The ORP1L isoform binds to the ER via its FFAT motif, and localizes to late endosomes by interacting with the small GTPase Rab7 [125,126]. ORP1L’s ORD domain not only binds sterols but also interacts with PI(4,5)P2 and PI(3,4)P2. These phosphoinositides allosterically enhance ORP1L-mediated cholesterol transport [127].

ORP5 and ORP8 possess a C-terminal transmembrane domain, which makes them ER-residing proteins. Du et al. reported that ORP5 facilitates the removal of cholesterol from the limiting membrane of late endosomes and lysosomes [128]. Subsequent studies suggested that ORP5 regulates PI4P levels in lipid droplets (LDs) through its role at ER-LD contacts [121,129]. The cell and in vitro liposome-based lipid transport experiments suggested that ORP5 and ORP8 can mediate the exchange of PS and PI4P/PI(4,5)P2 at ER-PM contact sites [130,131], and function at ER–mitochondria MCSs [132]. ORP3 is another OSBP/ORPs family member localized to ER-PM MCS. Unlike ORP5/ORP8, ORP3 has no transmembrane domain but can localize to ER via its FFAT motif. It has been reported that ORP3 regulates PI4P homeostasis and Ca^2+^ dynamics. [133,134,135]. ORP2 is a cytosolic LTP capable of transporting intracellular cholesterol and regulating cholesterol levels at the PM [136,137]. ORP6, as demonstrated by Mochizuki et al. [138], is involved in PI4P turnover at the ER-PM MCS of cerebellar granule neurons.

## 5. Conclusions

Reconstituted membrane systems have emerged as indispensable tools for uncovering the molecular mechanisms of intracellular membrane trafficking. By recapturing key cellular processes in vitro, these systems bridge the gap between structural studies and complex cellular environments, enabling explorations of the roles of proteins, lipids, and their interplay in membrane dynamics. They provide a simplified yet powerful platform to dissect complex biological processes that are difficult to study in their cellular environments.

The application of reconstituted membrane systems, including liposome-liposome, liposome-nanodisc lipid mixing or content mixing assays, single-vesicle fusion assays, and FRET-based lipid transport assays, has provided indispensable insights into the mechanisms of vesicle fusion and non-vesicular lipid transfer. These approaches have been particularly transformative in unraveling how SNAREs cooperate with other regulatory factors (e.g., synaptotagmin, Munc18, NSF, αSNAP) to achieve spatial and temporal regulation of membrane fusion. Similarly, the study of LTP-mediated lipid transport at MCSs has benefited tremendously from reconstituted membrane systems. The discovery and characterization of LTPs, including non-selective E-Syts, VPS13, and ATG2, as well as the lipid-selective OSBP/ORPs family proteins, have revealed their complicated mechanisms for maintaining lipid homeostasis and signal transduction at MCSs. The reconstituted membrane systems allow for the detailed examination of these LTPs’ lipid binding preference, transfer kinetics, and regulatory mechanisms, highlighting their diverse roles in cellular physiology. Recent studies revealed that phase-separated compartments play essential roles in organelle health and membrane behavior [139,140,141,142]. The membrane-associated phase separation is involved in various functional membrane organizations [143,144,145,146,147]. Our understanding of how membrane trafficking is regulated under phase separation is still at the beginning. The reconstituted membrane systems, such as GUVs, would be an ideal model to reveal the impacts of phase separation in organizing membrane compartments and regulating vesicle or non-vesicular trafficking.

Future studies may consider integrating additional layers of complexity, such as organelle-specific lipid compositions, cytoskeletal interactions, and post-translational modifications, into reconstituted systems. Advances in high-resolution imaging, microfluidics, and dynamic lipidomic profiling will further enhance the physiological relevance of these models. Moreover, the imbalances of membrane trafficking and lipid dysregulation are associated with many diseases, such as neurodegenerative diseases, metabolic disorders, and cancer. It will be critical to apply the mechanistic insights from reconstituted membrane systems to understand the in vivo physiologies and pathologies linked to intracellular membrane trafficking.

## Data Availability

No new data were created.
